# Long-term Cre-mediated retrograde tagging of neurons using a novel recombinant pseudorabies virus

**DOI:** 10.3389/fnana.2014.00086

**Published:** 2014-09-03

**Authors:** Hassana K. Oyibo, Petr Znamenskiy, Hysell V. Oviedo, Lynn W. Enquist, Anthony M. Zador

**Affiliations:** ^1^Watson School of Biological Sciences, Cold Spring Harbor Laboratory, Cold Spring HarborNY, USA; ^2^Friedrich Miescher Institute for Biomedical ResearchBasel, Switzerland; ^3^Biozentrum, University of BaselBasel, Switzerland; ^4^Department of Biology, City College of New YorkNew York, NY, USA; ^5^Molecular Biology and Princeton Neuroscience Institute, Princeton UniversityPrinceton, NJ, USA

**Keywords:** pseudorabies virus, retrograde tracing, viral tracing, immediate early gene (IEG), auditory cortex, non-toxic retrograde labeling, secondary visual cortex

## Abstract

Brain regions contain diverse populations of neurons that project to different long-range targets. The study of these subpopulations in circuit function and behavior requires a toolkit to characterize and manipulate their activity *in vivo*. We have developed a novel set of reagents based on Pseudorabies Virus (PRV) for efficient and long-term genetic tagging of neurons based on their projection targets. By deleting IE180, the master transcriptional regulator in the PRV genome, we have produced a mutant virus capable of infection and transgene expression in neurons but unable to replicate in or spread from those neurons. IE180-null mutants showed no cytotoxicity, and infected neurons exhibited normal physiological function more than 45 days after infection, indicating the utility of these engineered viruses for chronic experiments. To enable rapid and convenient construction of novel IE180-null recombinants, we engineered a bacterial artificial chromosome (BAC) shuttle-vector system for moving new constructs into the PRV IE180-null genome. Using this system we generated an IE180-null recombinant virus expressing the site-specific recombinase Cre. This Cre-expressing virus (PRV-hSyn-Cre) efficiently and robustly infects neurons *in vivo* and activates transgene expression from Cre-dependent vectors in local and retrograde projecting populations of neurons in the mouse. We also generated an assortment of recombinant viruses expressing fluorescent proteins (mCherry, EGFP, ECFP). These viruses exhibit long-term labeling of neurons *in vitro* but transient labeling *in vivo*. Together these novel IE180-null PRV reagents expand the toolkit for targeted gene expression in the brain, facilitating functional dissection of neuronal circuits *in vivo*.

## Introduction

Neuroinvasive viruses have become an essential part of the systems neuroscience toolkit as a vehicle for delivery of genetic material into neurons. Their applications range from anatomical tracing with fluorescent proteins to monitoring and manipulating the activity of defined populations of cells (Boyden et al., 2005; Deisseroth, 2011; Sasaki et al., 2011; Akerboom et al., 2012). Viral approaches can be used to deliver transgenes into species in which it is not possible or convenient to produce transgenic animals. Additionally, targeted viral injections can label anatomically restricted populations of neurons.

One powerful approach to labeling neuronal subpopulations exploits the ability of certain viruses to infect axon terminals and undergo retrograde transport to the soma, thereby labeling neurons that project to the injection site. This approach can target populations of projection neurons without the need for a cell-type specific promoter to direct gene expression. However, the utility of existing viral systems for retrograde labeling is limited by a number of restrictions, including infection inefficiency, unpredictable tropism, cytotoxicity, limited payload capacity, safety concerns and/or unavailability. Our goal was to produce a set of retrograde viral tools that would be suitable not only for the mapping of connectivity, but for the long-term manipulation of specific anatomically and genetically defined neuronal populations.

PRV is a large double stranded DNA virus in the alphaherpesvirinae subfamily of herpesviridae. Because PRV efficiently labels local and retrograde populations and is transmitted transynaptically, it has been used widely for polysynaptic circuit tracing (Strack and Loewy, 1990; Kim et al., 1999; Yang et al., 1999; Aston-Jones and Card, 2000; Smith et al., 2000; Card and Enquist, 2001; Horváth et al., 2002; Yoon et al., 2005; Willhite et al., 2006; Campbell and Herbison, 2007; Boldogkoi et al., 2009; Braz et al., 2009; Ohara et al., 2009a,b). However infection with PRV is cytotoxic, killing cells in days or even hours depending on the strain and titer of infection and is eventually lethal to the animal. To restrict PRV labeling to first order neurons and eliminate its toxicity, we took advantage of the fact that PRV has only one immediate early gene, IE180, which acts as the master switch of the PRV transcriptional cascade (Ihara and Ben-Porat, 1985). By deleting IE180, we blocked the viral replication cycle, thereby eliminating PRV cytotoxicity, viral particle production, and transsynaptic spread. Here we show that IE180-null mutants grown in an IE180 expressing cell line are capable of infecting neurons and undergo retrograde transport in the central nervous system. One mutant, PRV-hSyn-CRE, is capable of efficiently labeling even sparse subpopulations of neurons projecting to the site of injection in a reporter mouse. Furthermore, IE180-null PRV-infected neurons persist for months after injection and are physiologically indistinguishable from uninfected cells. Unlike other retrograde viruses such as rabies virus (RV) and herpes simplex virus 1 (HSV-1), PRV is not a human pathogen and is safer and more convenient to work with (Kluge et al., 1999; Pomeranz et al., 2005). The IE180-null PRV system described here represents a novel set of reagents useful for both anatomical and functional retrograde circuit mapping and analysis.

## Methods

The PRV Becker bacterial artificial chromosome (BAC) vector (pBecker3) described previously (Smith and Enquist, 2000) was modified using a λ-mediated counter selection BAC recombination system from Gene Bridges (Gene Bridges #K002) (Muyrers et al., 1999). We first synthesized a plasmid containing two FRT sites flanking a multiple cloning site (IDT Inc.). An rpsL-zeo positive/negative selection cassette was then subcloned into the multiple cloning site (MCS) yielding the vector PZ26 (FRT-Neo-rpsL-FRT). PZ26 was amplified with primers containing homology to IE180 (CGGCCCGCCAGAGAAGAGTCTTCTTCTTCTCCTCCTCCGGCCGCCTTCCTCCTTCTTCTCGGAATTCGCCCTTAACCATATG and CAAACTCTTTTCTCACCCGATGGGAGAAGGAGGAGAAGGGGACCGGGGGACCGCGGGAGGGCCAGTGTGGATGGATATCTGGCAG). There are two copies of the PRV IE180 gene, one copy in each of the large inverted repeats surrounding the unique short region of the genome. After replacing the first copy of IE180 with the FRT-rpsL-Zeo-FRT cassette, we transformed the BAC clone with 707-FLPe (Gene Bridges) and induced the expression of Flp recombinase to excise the cassette leaving behind one FRT site. BACs that successfully excised the cassette were selected on streptomycin and verified by diagnostic digest. These steps were repeated to remove the second copy of IE180 yielding a PRV viral BAC with FRT sites replacing both copies of IE180 (PZ42).

To make an IE180- and Us9- null pBecker (PZ57) BAC, we used recombineering to replace Us9 in PZ42 with a neo-rpsL cassette (Gene Bridges) amplified with Us9 homology arms (primers GGAGAACCGGCCCGCCCGCATTCCGACATGCCCGCCGCCGCCCCCGCCGACATGGACACGGGCCTGGTGATGATGGCGGGATCG and GCGGCGGGGCGGGCGGCCACCACCCGCTCGCTACACGTGCCGGGCGATGATGCCCCCGATTCAGAAGAACTCGTCAAGAAGGCG). Positive clones were selected on kanamycin plates and verified by diagnostic digest. The shuttle vector was constructed by subcloning the em7 (promoter)-zeocin cassette into a minigene containing a multiple cloning site flanked by homology arms matching the rpsL sequence, which was synthesized by IDT [HA–MCS–HA (GGCCTGGTGATGATGGCGGGATCGTTGTATATTTC TTGACACCTTTTCGGCATCGCCCTAAAATTCGGCGTCCTCATATTGTGTGAGGACGTTTTATTAC—AACGTTACGCGTAAGCTTCTAGAATTCATTAATGCATGC–GGCGGCGAATGGGCTGACCGCTTCCTCGTGCTTTACGGTATCGCCGCTCCCGATTCGCAGCGCATCGCCTTCTATCGCCTTCTTGACGAGTTCTTCTGA)]. Transgenes were subcloned into the MCS of the shuttle vector and the recombination cassette was amplified using the shuttle forward (GGCCTGGTGATGATGGCGGGATCGTTG) and shuttle reverse (TCAGAAGAACTCGTCAAGAAGGCGATAGAAG) primers. Transgenes constructs cloned into this BAC include the synapsin promoter expressing Cre (hSyn-Cre), and the elongation factor 1a (*EF1a*) promoter driving expression of Green Fluorescent Protein, mCherry or Cyan fluorescence protein (*EF1a*-EGFP, *EF1a*-mCherry and *EF1a*-ECFP).

### Inducible IE180 expression cell line

As previous work had indicated that constitutive expression of IE180 is toxic to cells (Taharaguchi et al., 2003), we constructed the cell line PK15-IE180, which has conditional IE180 expression. We made a cell line with the reverse tetracycline-controlled transactivator (rtTA) and then inserted a transgene under the control of the tetracycling-inducible promoter, pTRE (promoter tetracycline response element) (Gossen et al., 1995). We inserted the rtTA sequence into PK15 cells by infecting with the rtTA-hygro Murine Stem Cell Virus (MSCV) retrovirus and selecting for transduced cells with hygromycin, yielding the cell line PK15-rtTA. We next used BAC recombineering to subclone IE180 from pBecker3 into the pUC19 cloning vector (Smith and Enquist, 2000). IE180 was then excised from this vector and subcloned into the retroviral vector under the control of the rtTA-regulated pTRE promoter pTREtight (TTiGP). PK15-rtTA cells were then infected with TTiGP-IE180 retrovirus and plated at clonal density in the presence of puromycin to select positive clones. Individual clones were then tested by transfection of PZ42 IE180-null BAC in the presence of 2 μg/ml doxycycline. The clone that produced the highest titers was selected, expanded, and used for making stocks of IE180-null mutants.

### Virus production

The initial production of viruses from PRV BACs is accomplished by transfection of BAC DNA prepared from 1 ml of an overnight culture into a 35 mm dish of PK15-IE180 cells by Lipofectamine 2000 (Life Technologies). Immediately after transfection 2 μg/ml doxycycline was added to the media. When ~80% of cells exhibited cytopathic effects (typically 2–4 days after transfection) the virus stock was harvested, and used to infect a larger volume of cells. Subsequently, new batches of viral stocks were prepared by amplifying them in PK15-IE180 cells. Virions were concentrated and titered as previously described (Card and Enquist, 2001) on PK15-IE180 cells in the presence of 2 μg/ml doxycycline in the culture media.

### Dissociated neuronal culture

Mouse cortical and hippocampal tissue was acquired from BrainBits (BrainBits® LLC, Springfield, IL). The tissue was dissociated and plated at approximately 16,000 cells/cm^2^ in poly-D-lysine coated tissue culture plates (BrainBits standard protocol^1^). During plating in NbActiv1 media, neurons were infected with PRV HKO128 at a multiplicity of infection (MOI) of 10 and incubated at 37°C. Six hours after plating 100% of the media was replaced. Thereafter, 50% of the media was exchanged for new NbActiv1 media every 3 days.

### Animal care and viral injections

All animal protocols were approved by the Cold Spring Harbor Laboratory Animal Care and Use Committee and carried out in accordance with National Institutes of Health standards. Mice were anesthetized with a mixture of ketamine/medetomidine (120 mg/kg ketamine, 0.5 mg/kg medetomidine). For each injection a small craniotomy was made above the injection site and a glass micropipette was used to deliver ~250 nl of the virus. Injections were performed in mice of either sex by delivering brief pulses of pressure at 0.2 Hz using a Picospritzer II (Parker), each pulse delivering ~2 nl. Ai14 mice (LSL-tdTomato, JAX labs) were injected with PRV-hSyn-CRE IE180-null PRV (titer 3.0 × 10^9^ pfu) in left auditory cortex (2.5 mm posterior to bregma, 4.5 mm lateral from midline) with a single injection at a depth of 500 μm from the pia. ~250 nl of PRV *EF1a*-EGFP, PRV- *EF1a*-Mcherry and PRV-*EF1a*-ECFP were injected at titers of ~7 × 10^8^ pfu.

### Histology and confocal imaging

Mice were deeply anesthetized with ketamine/medetomidine and transcardially perfused with 0.15 M NaCl followed by 4% paraformaldehyde (PFA). The brains were removed and post-fixed overnight in 4% PFA before being sectioned into 100 μm slices. Slices were mounted in VectaShield with DAPI (Vector Laboratories) and imaged using the LSM710 confocal microscope (Zeiss). Z-stacks spanning 50 μm were acquired of the entire brain slices or regions of interest. For presentation of the distribution of labeled neurons, maximum intensity projection images were calculated using ImageJ (NIH) software.

### Slice preparation and electrophysiology

Ai14 mice were injected with PRV-hSyn-Cre in the left auditory cortex at postnatal day 30. At 40–70 days post-injection, animals were anesthetized with isofluorane and perfused intracardially with cold artificial cerebrospinal fluid (ACSF) containing 127 mM NaCl, 25 mM NaHCO_3_, 25 mM D-glucose, 2.5 mM KCl, 1 mM MgCl_2_, 2 mM CaCl_2_, and 1.25 mM NaH_2_PO_4_, aerated with 95% O_2_ 5% CO_2_. The brains were transferred to a chilled slicing solution composed of 110 mM choline chloride, 25 mM NaHCO_3_, 25 mM D-glucose, 11.6 mM sodium ascorbate, 7 mM MgCl_2_, 3.1 mM sodium pyruvate, 2.5 mM KCl, 1.25 mM NaH_2_PO_4_, and 0.5 mM CaCl_2_. We made 300 μm thick horizontal slices that were incubated in ACSF at 34° for 20–30 min and then kept at room temperature during the experiments.

To examine the physiological properties of retrogradely labeled neurons in the right auditory cortex, we made current-clamp recordings of excitatory neurons in layers 3 and 5 45–80 μm below the surface of the slice. Neurons were patched with electrodes (4–6 MΩ) containing intracellular solution (128 mM K-methylsulfate, 4 mM MgCl_2_, 10 mM HEPES, 1 mM EGTA, 4 mM Na_2_ATP, 0.4 mM Na_2_GTP, 10 mM Na-phosphocreatine, and 0.015 mM Alexa-594 (Molecular Probes, Eugene, Oregon, USA), pH 7.25, 300 mOsm). The membrane resting potential and input resistances reported were all measured in whole-cell configuration immediately after break-in. Cells were included in analysis if their resting membrane potential did not become depolarized during the recording, and series resistance did not increase above 35 MΩ. Statistical comparisons were done using Wilcoxon sign-rank test comparing pairs of nearby PRV-infected and control neurons.

### *In vivo* physiology

Two FVB mice were injected with AAV-*EF1a*-DIO-hChR2 (H134R)-EYFP supplied from UNC vector core. 2–4 weeks after injection, the mice were anesthetized using a mixture of ketamine/medetomidine (120 mg/kg ketamine, 0.5 mg/kg medetomidine) and placed in an orbitonasal stereotaxic apparatus. A cranial window ~2 mm in diameter was made to expose the left primary auditory cortex and the dura was resected. For light stimulation, 1 ms pulses of blue light at 10 mW from a 473 nm laser were projected on the cortical surface. Spike responses were recorded extracellularly using acute NeuroNexus probes. Signals were amplified, filtered 600–9000 Hz and acquired using the Cheetah 32 data acquisition system (Neuralynx). Single units were identified after spike sorting using MClust software.

## Results

### Production and validation of IE180-null PRV

Herpes viruses have three phases of gene expression: immediate early (IE), early (E) and late (L). IE gene expression occurs in the absence of new protein synthesis and is activated by viral transactivators that enter the cell in the viral tegument, a proteinaceous layer between the capsid and envelope. IE genes are required for E gene expression, whose gene products promote the replication of viral DNA. Newly replicated DNA is the substrate for L gene expression. Herpes Simplex Virus-1 (HSV-1) encodes five immediate early genes, and the recombinant HSV-1 used for retrograde tagging has deletions in two of the essential IE genes; ICP4 and ICP27, and also carries a mutation in the tegument protein VP16 that blocks transactivation of the other 3 IE genes (Lilley et al., 2001). The single PRV IE gene, IE180, acts as the master transcriptional regulator of PRV gene expression. We reasoned that elimination of the IE180 gene should completely prevent PRV replication and spread.

**Figure 1:**
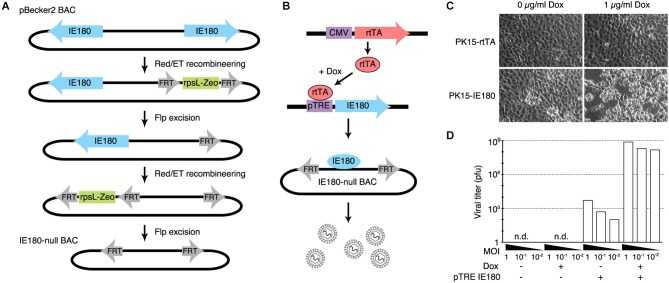
**Design and construction of the IE180-null PRV mutant. (A)** Recombineering strategy for deletion of both copies of *IE180* from the PRV pBecker3 BAC carrying the PRV genome. **(B)** Inducible IE180 trans-complementation system for packaging IE180-null PRV. To make the PK15-IE180 cell line we first stably expressed the reverse Tet-transactivator rtTA in PK15 cells. We then inserted IE180 under the control of the Tet regulated pTreTIGt promoter. The addition of doxycycline to the cell growth media results in IE180 expression and thus viral production. **(C)** PK15-rtTA cells and PK15-IE180 cells were infected with IE180-null PRV and imaged 48 h later. Cytopathic effects associated with PRV replication were observed only in PK15-IE180 cells and were enhanced by the addition of doxycycline. **(D)** Titers of viral supernatants produced by PK15 and PK15-IE180 cells infected with IE180-null PRV (harvested 3 days after infection) at MOI of 1, 0.1 or 0.01 quantified on PK15-IE180 cells. No infectious material can be detected in supernatants of PK15 cultures lacking IE180.

The PRV genome is ~143 kb and consists of two long inverted repeat regions flanking a shorter unique region. The gene encoding IE180 is present in duplicate; one in each of the inverted repeat regions. We used BAC recombineering methods to delete both copies of the IE180 open reading frame (ORF) from pBecker3, a self-excising PRV BAC carrying the PRV genome (Smith and Enquist, 2000; Figure 1A). Transfection of this BAC into PK15 cells produced no infectious viral particles detectable by plaque assay, showing that deletion of IE180 was sufficient to abolish viral replication and packaging.

To enable construction and propagation of IE180-null mutants, we developed a method to express IE180 in *trans*. Co-transfection of the IE180–null BAC with an IE180 expression plasmid into PK15 cells resulted in recombination events between the plasmid and BAC that produced replication competent, IE180-expressing virus. We therefore constructed a cell line that carried a genomic cassette for conditional expression of IE180, since this genomic IE180 would be less likely to undergo recombination. The cell line was derived from PK15 cells that efficiently replicate PRV. Because previous work suggested that constitutive expression of IE180 is toxic (Taharaguchi et al., 2003), we placed IE180 under the control of a tetracycline inducible promoter (PK15-IE180 cells, Figure 1B; Gossen et al., 1995).

To validate the replication deficient phenotype of the IE180 deleted PRV BAC and the complementation efficiency of the PK15-IE180 cell line, we first transfected IE180-null viral BAC DNA into PK15-rtTA or PK15-IE180 cells in the presence or absence of doxycycline (Figure 1C). Cytotoxic effects were observed only in transfected PK15-IE180 cells and infectious virions were produced only from this cell line. These experiments demonstrated the requirement of IE180 expression for the production of infectious virus from the IE180-null PRV BAC (Figure 1C). Since BAC transfections are often inefficient and variable, we next tested the requirement of IE180 for viral propagation during serial viral passaging. We harvested the virions produced by transfection of the IE180-null BAC into PK15-IE180 cells and used this stock to infect PK15 or PK15-IE180 cells in the presence or absence of doxycycline. We collected the supernatants from each culture and titered infectious material by plaque assay on PK15-IE180 cells. No plaques were produced from the supernatants harvested from PK15 cells (detection threshold of 10^3^ pfu/ml) (Figure 1D). On the other hand, while some pfus were generated from the supernatants of PK15-IE180 cells in the absence of doxycycline, the addition of doxycycline increased the resulting viral titers 105–106 fold (Figure 1D). Infectious virus produced from the PK15-E180 cell line in the absence of doxycycline likely reflects low level leak expression of IE180 from the TTiGP promoter. However, such expression does not appear to be cytotoxic and does not interfere with the propagation of PK15-IE180 cells which retain the ability to produce high titer PRV stocks after as many as 20 passages. After the initial transfection, IE180-null mutants can be propagated and amplified in PK15-IE180 cells and concentrated to produce titers exceeding 10^9^ pfu/ml. Together these results indicate that deletion of IE180 from the genome of PRV blocks production of infectious virus particles and that high titer stocks of IE180-null PRV mutants can be produced when IE180 is complemented *in trans*.

### Insertion of transgenes into IE180-null PRV mutants

**Figure 2:**
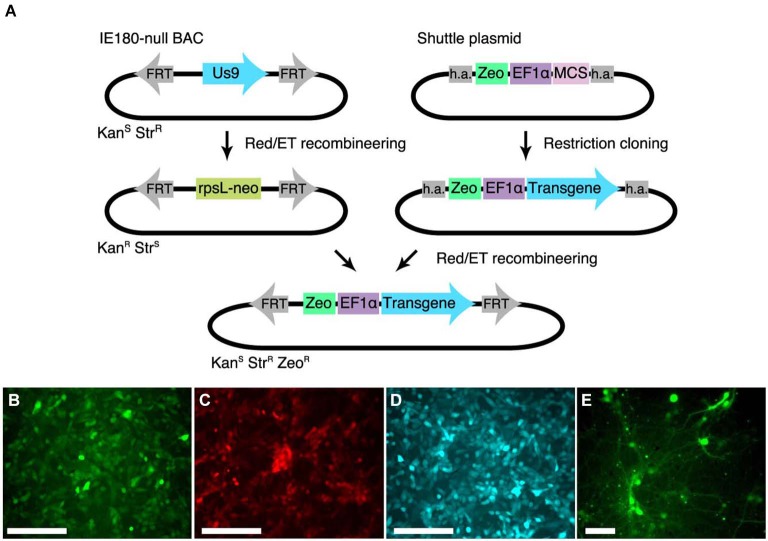
**Shuttle vector and landing pad system for streamlining IE180-null PRV mutant recombinants. (A)** A landing pad was inserted into the PRV IE180-null BAC. The transgene is inserted into a shuttle plasmid containing homology arms (h.a.) to the landing pad. The transgene is then amplified together with the Zeocin selection cassette using standardized primers and inserted into the IE180-null BAC using recombineering. **(B–D)** PK15 cells infected with PRV strains expressing EGFP (PRV HKO128), mCherry (PRV HKO242) and ECFP (PRV HKO243). **(E)** Dissociated neurons infected with PRV HKO128, at 3 weeks post infection.

To streamline the construction of recombinant PRV in the IE180-null background, we developed a shuttle system for reliable insertion of transgenes into the IE180-null PRV BAC. We first inserted a recombineering landing pad, carrying an rspL-kan^R^/neo^R^ selection cassette that confers resistance to kanamycin and sensitivity to streptomycin into the IE180-null PRV genome disrupting the Us9 locus. Us9 is required for anterograde viral spread *in vivo* (i.e., spread from presynaptic to postsynaptic neurons) but its deletion has no effect on primary infection properties of PRV (Lyman et al., 2007). We then constructed a shuttle vector with a Zeocin resistance cassette and a MCS flanked by homology to the landing pad (Figure 2A). The sequence flanking the selection cassette is specific to the landing pad inserted into Us9 and lacks homology to any other region in the viral genome, which improves specificity of recombination. Transgenes can be inserted readily into the MCS using standard cloning techniques. The shuttle cassette can then be isolated by PCR amplification or restriction digestion and used to deliver the transgene into the PRV IE180-null BAC by recombineering. Successfully modified BAC clones can then be identified as zeocin- and streptomycin-resistant and neomycin-sensitive.

To test expression of transgenes in the PRV IE180-null background, we used the shuttle system to produce several recombinants carrying transgenes encoding different fluorescent proteins. We used the constitutive *Ef1a* promoter to drive expression of EGFP (PRV HKO128), mCherry (PRV HKO242) and ECFP (PRV HKO243).

These viruses efficiently drove fluorescent protein expression in PK15 cells (Figures 2B–D) and dissociated neuronal cultures (Figure 2E). Surprisingly, *in vivo* injection of PRV HKO128 (expressing EGFP) into auditory cortex labeled only neurons near the injection site but did not label input structures such as the contralateral auditory cortex or the medial geniculate body (MGB) (*not shown*). Injection into other structures, including auditory striatium and cerebellum, also failed to label input structures. Thus in their current form, these reagents are useful only for *in vitro* experiments (see Section Discussion).

### Gene expression from IE180-null PRV mutants *in vivo*

We hypothesized that the *in vivo* expression of fluorescent proteins from the recombinants we had generated was either too weak or too transient to be useful *in vivo*. However, because even weak and transient expression of Cre recombinase can effectively activate gene expression, we expected that an IE180-null PRV expressing Cre recombinase would be a useful reagent.

To produce a PRV IE180-null recombinant that could be used in combination with Cre-dependent viruses and Cre-reporter mice that are already a widely used component of the systems neuroscience toolkit, we inserted the Cre-recombinase gene under control of the neuron specific synapsin-1 promoter (hSyn)(Kügler et al., 2003).

**Figure 3:**
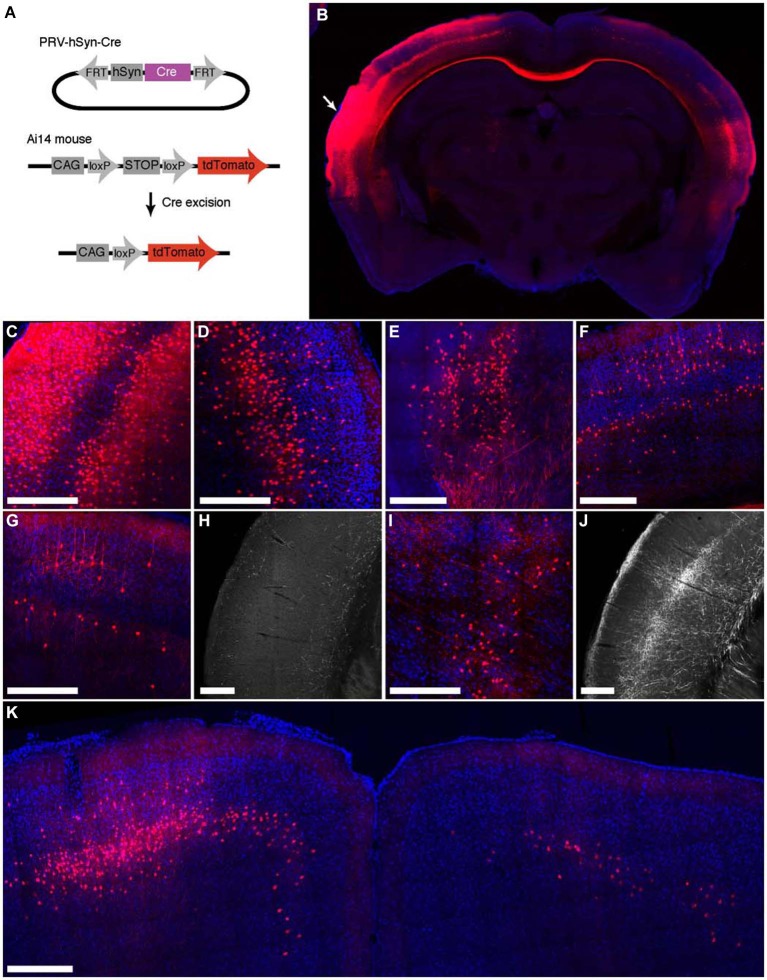
**Retrograde labeling by PRV IE180-null-hSyn-Cre in primary auditory cortex. (A)** PRV-hSyn-Cre activates tdTomato expression in Ai14 reporter mice. **(B)** Fluorescence image showing the distribution of tdTomato labeling in an Ai14 mouse injected with PRV-hSyn-Cre in the primary auditory cortex. Arrow points to site of injection. **(B–G,I,K)** Retrograde labeling by PRV-hSyn-Cre in **(C)** the auditory cortex ipsilateral to the site of injection, **(D)** contralateral auditory cortex, **(E)** medial geniculate body, **(F)** ipsilateral parietal cortex, **(G)** contralateral parietal cortex, **(I)** the lateral posterior and the posterior nuclei in the thalamus and **(K)** motor cortex. **(H)** Axonal labeling in the primary auditory cortex of a mouse injected with AAV-EGFP in the contralateral parietal cortex, adapted from Allen Mouse Brain Connectivity Atlas. **(J)** Axonal labeling in the primary auditory cortex of a mouse injected with AAV-EGFP in the ipsilateral posterior and lateral posterior nuclei. Scale bars—300 um.

To characterize the ability of IE180-null PRV-hSyn-Cre virus for retrograde infection after injection in the brain, we injected it into Ai14 mice, which carry a Cre-dependent tdTomato expression cassette inserted at the ROSA26 locus (Madisen et al., 2010; Figure 3A). The distribution of tdTomato fluorescence 4 months after injection into auditory cortex is shown in Figure 3. In addition to the site of injection (Figures 3B,C), fluorescent neurons were found in known input structures of the auditory cortex, including the contralateral auditory cortex (Figures 3B,D; Roger and Arnault, 1989; Oviedo et al., 2010), MGB in the thalamus (Figure 3E; Roger and Arnault, 1989; Pandya, 1995; Lima et al., 2009), the ipsilateral posterior parietal cortex (Figure 3F; Reep et al., 1994; Pandya, 1995) as well as the ipsilateral motor cortex (Figure 3K; Nelson et al., 2013). Labeled neurons in contralateral auditory cortex were found primarily in layers 3–5, the established origin of the callossal projection in the mouse auditory cortex (Oviedo et al., 2010).

We also observed sparse tdTomato labeling in regions, for which projections to the auditory cortex have not been previously described, including the contralateral posterior parietal cortex (Figure 3G), the contralateral motor cortex (M1, M2, Figure 3K) and the posterior and the lateral posterior nuclei in the thalamus (Figure 3I; Roger and Arnault, 1989; Nelson et al., 2013). To validate the existence of these projections, we examined anterograde labeling data from these areas (collected as a part of the Allen Mouse Brain Connectivity Atlas) to test for projections to the primary auditory cortex. We found sparse axonal labeling in the primary auditory cortex in mice injected with AAV-EGFP in contralateral posterior parietal cortex (Figure 3H, ^2^ (Allen Institute for Brain Science, 2013a) as well as the ipsilateral posterior thalamic nucleus (Figure 3J; Allen Institute for Brain Science, 2013b), confirming the existence of direct inputs from these areas into auditory cortex. Retrogradely labeled neurons were also found in zona incerta and nucleus basalis, regions known to project widely in the neocortex (Mitrofanis, 2005), as well as in the ventral division of the basolateral amygdala (not shown). These observations illustrate the broad tropism IE180-null PRV and its ability to robustly label even exceedingly sparse projections.

**Figure 4:**
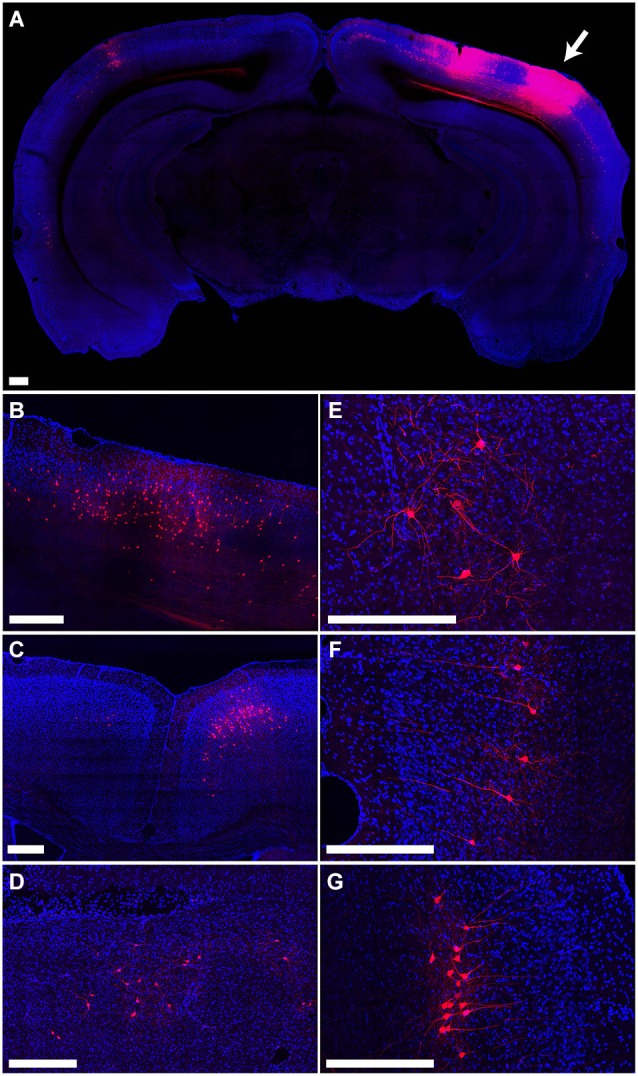
**Retrograde labeling by PRV IE180-null-hSyn-Cre in secondary visual cortex. (A)** Fluorescence image showing the distribution of tdTomato labeling in an Ai14 mouse injected with PRV-hSyn-Cre in the secondary visual cortex. Arrow points to site of injection. **(B–G)** Retrograde labeling by PRV-hSyn-Cre in **(B)** primary visual cortex ipsilateral to the site of injection, **(C)** anterior cingulate cortex, **(D)** lateral posterior nucleus of the thalamus, **(E)** ipsilateral amygdala, **(F)** contralateral perirhinal cortex, **(G)** ipsilateral perirhinal cortex. Scale bars—300 um.

The distribution of tdTomato labeling 15 days after injection of PRV-hSyn-Cre into the secondary visual cortex area AL of an Ai14 mouse is shown in Figure 4. This injection resulted in labeling of cells at the site of injection (Figure 4A) as well as regions known to project to area AL, including primary visual cortex (Figure 4B), primary and secondary auditory cortex, contralateral visual cortex (Figure 4A), anterior cingulate and secondary motor cortex (Figure 4C), the ipsilateral LP (Figure 4D), VA/VL and posterior nuclei in the thalamus, the ipsilateral (Figure 4E) and contralateral amygdala, and the ipsilateral and contralateral perirhinal cortex (Figures 4F,G).

### Viability and intrinsic properties of infected neurons

The application of PRV in the study of neural circuits has been limited by its rapid cytotoxicity; wild-type PRV alters neuronal properties within hours of infection and cell death follows shortly thereafter (McCarthy et al., 2009). Since IE180 acts as the master transcriptional regulator of PRV gene expression, its deletion should not only prevent replication, production of viral particles, and spread of infection, but should also eliminate the resultant cytotoxic effects. We found that Ai14 mice injected with PRV-hSyn-Cre showed no overt signs of infection even after 6 months of infection (*n* = 3 mice). Moreover, tdTomato expressing neurons were observed at the earliest tested time point of 3 days after infection (*n* = 3 mice) and persisted as long as 6 months (the longest time point examined, *n* = 3 mice).

**Figure 5:**
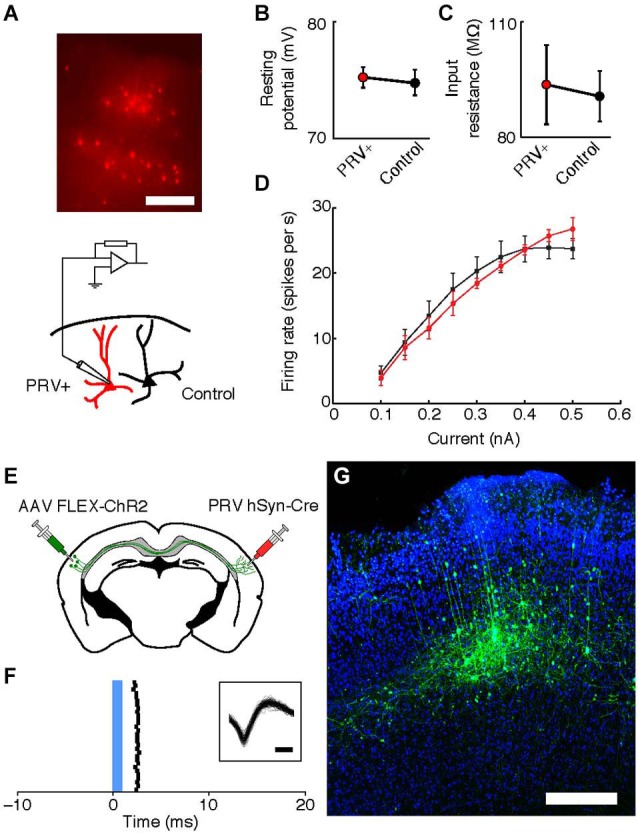
**Intrinsic properties of PRV IE180-null hSyn-Cre infected neurons are indistinguishable from control cells 40–70 days after infection. (A)** tdTomato fluorescence in neurons retrogradely labeled with PRV IE180-null hSyn-CRE in an acute slice of Auditory cortex before recording. Scale bar ~200 um. **(B)** Resting potential is unaffected in PRV infected neurons (*p* = 0.7266). **(C)** Input resistance is unaffected in PRV infected neurons (*p* = 0.8750). **(D)** F/I (firing rate/current) curves are unchanged by PRV infection (*p* = 0.7153). **(E)** Strategy for ChR2-tagging of callosal projection neuron in Auditory cortex using PRV-hSyn-Cre. **(F)** Raster of light-evoked responses of a presumed callosal projection neuron. Blue bar- light stimulus. Inset shows extracellular spike waveforms of this neuron, bar—200 us. **(G)** ChR2-YFP expression in PRV-tagged callosal projection neurons in the auditory cortex. AAV-*EF1a*-DIO-hChR2 (H134R)-EYFP was injected into the left auditory cortex and IE180-null PRV-hSyn-Cre was injected into the right auditory cortex, shown here is the left auditory cortex with callosal projection neurons labeled as a result of dual infection. Scale bar—300 um.

To determine the physiological health of cells infected with PRV-hSyn-Cre we performed slice recordings of retrogradely-infected neurons in the auditory cortex contralateral to the site of infection in Ai14 mice. We measured the resting potential, input resistance and f/I (firing rate/current) curves of neurons 40–70 days after infection (mean = 48.75 days, ±14.2). We found that the intrinsic properties of IE180-null PRV infected cells (*n* = 9 pairs in 4 animals) were indistinguishable from adjacent uninfected control cells (Figures 5A–D, mean distance between pairs = 37.1 ± 31.9 μm.

### PINPing with PRV

One possible application of IE180-null PRV mutants is the expression of ligand or light-gated ion channels in neurons based on the target of their projections. This would allow application of the Photostimulation-assisted Identification of Neuronal Populations (PINP) technique (Lima et al., 2009), wherein populations of neurons defined by their projection pattern are identified during electrophysiological recordings. To explore the potential of IE180-null PRV-hSyn-Cre in such experiments, we tested its ability to induce ChR2 expression from an adeno-associated virus (AAV) carrying a Cre-dependent FLEX-ChR2-YFP expression cassette (*n* = 2 mice). When AAV FLEX-ChR2-YFP was injected into the left auditory cortex and PRV-hSyn-Cre in the right auditory cortex (Figure 5E), ChR2-YFP fluorescence was confined to layers 3–5 of the left auditory cortex (Figure 5G), the origin of the contralateral projection in mouse auditory cortex (Oviedo et al., 2010). Neurons, which responded to brief flashes of blue light, could be identified during *in vivo* extracellular recordings from injected mice (Figure 5F). Together, these results illustrate the utility of IE180-null PRV mutants as agents for specific manipulation of neurons based on the targets of their projections.

## Discussion

By deleting IE180, the master transcriptional regulator in the PRV genome, we have produced PRV mutants capable of long-term labeling of local and retrograde neuronal populations. We have engineered a rapid and convenient system for the construction of novel IE180-null PRV recombinants. Using this system we made an assortment of recombinant viruses expressing the fluorescent proteins mCherry, EGFP, and ECFP as well as the site-specific recombinase Cre. The Cre-expressing virus, PRV-hSyn-Cre is a versatile tool capable of efficient and robust labeling of neurons *in vivo* through the activation of transgene expression from Cre-dependent constructs in transgenic mice or introduced via other viral vectors. These IE180-null PRV reagents expand the toolkit for targeted gene expression in the brain, facilitating functional dissection of neuronal circuits *in vivo*. Several viral vectors, including RV, AAVs, lentiviruses and canine adenovirus, have been used as vehicles for retrograde transgene expression. However, many of these reagents are limited by pathway tropisms, efficiency of retrograde transport or cytotoxicity. RV is widely used for its ability to spread transynaptically between neurons, enabling its use as a multisynaptic or monosynaptic tracer, but its applications are limited by its cytotoxicity (Ugolini, 1995; Wickersham et al., 2007; Ohara et al., 2009a). Recombinant AAV, which are the most widely used viral vectors for delivering transgenes into the brain, show some capacity for retrograde spread (Burger et al., 2004; Kaspar et al., 2004). However, their retrograde transport is often inefficient (*but see* Aronoff et al., 2010; Nelson et al., 2013) and limited to specific projection pathways. For example, AAV5 undergoes retrograde transport to the entorhinal cortex after injection into the dentate gyrus but fails to label extensive callosal inputs to the auditory cortex (Aschauer et al., 2013). Lentiviral vectors can be pseudotyped with the glycoproteins of different viruses to enable retrograde infection (Carpentier et al., 2011; Kato et al., 2011; Hirano et al., 2013), but these are often inefficient, yield poor expression, and suffer from pathway-specific tropism. Recombinant canine adenoviruses (CAV-2) have also been manipulated for long-term retrograde labeling of neuronal populations (Soudais et al., 2004), but again there is evidence for pathway-specific tropism (Senn et al., 2014). The tropisms and other limitations of current viral vectors for non-lethal retrograde labeling highlight the need for a diverse set of viral tools.

HSV-1 has been used for retrograde targeting of neuronal populations in long-term electrophysiological and behavioral experiments (Lilley et al., 2001; Lima et al., 2009; Znamenskiy and Zador, 2013). Unfortunately, this particular HSV-1 mutant is a patented and proprietary virus that is no longer available for academic use. Inspired by this work, we pursued a conceptually similar strategy in modifying Pseudorabies virus (PRV). Wild-type and attenuated strains of PRV are widely used in neuroanatomical studies for their ability to infect neurons retrogradely and spread between synaptically connected cells (Kim et al., 1999; Yang et al., 1999; Brideau et al., 2000; Card and Enquist, 2001, 2012). These properties make PRV useful as a transsynaptic tracer, labeling connected populations of neurons throughout the brain within days of infection. However, infection with wild-type or attenuated PRV results in physiological abnormalities in the infected neurons within hours and eventual death of the host, preventing its application in behavioral or physiological experiments requiring long-term cell viability (Aston-Jones and Card, 2000; McCarthy et al., 2009).

PRV mutants deficient in transneuronal spread have been constructed by eliminating the expression of viral genes encoding glycoprotein B (gB) required for virion infection and cell-cell spread, or thymidine kinase (TK) required for viral DNA synthesis in terminally differentiated cells (DeFalco et al., 2001; Curanovic and Enquist, 2009). However, while the deletion of these genes inhibits viral replication (TK) or spread (gB), neither deletion fully eliminates expression of native viral genes. Consequently, TK/gB-null viruses retain cytotoxicity and are not suitable for experiments that require long term cell survival.

Here we describe the production of a new set of reagents based on IE180-null PRV mutants, for targeted long-term genetic tagging of neurons in the brain. By deleting IE180, the singular master transcriptional regulator of PRV, we eliminated viral replication and cytotoxicity without affecting the capacity of IE180 complemented PRV particles for retrograde axonal transport. IE180-null PRV mutants efficiently transduce neurons *in vivo*, undergo retrograde transport, and efficiently label neurons for at least several months after CNS injection. Furthermore, infected neurons exhibit normal intrinsic physiological properties more than a month after infection, demonstrating that IE180-null PRV mutants do not perturb cellular physiology. Together, these properties make IE180-null PRV mutants suitable for not only anatomical tracing studies, but also experiments requiring long-term labeling.

To facilitate the construction of recombinant IE180-null PRV mutants, we used a shuttle-vector based strategy that avoids many of the pitfalls of BAC recombineering and reliably yields recombinant IE180-null PRV mutants carrying transgenes of interest with a turnaround time of ~13 days (4 days to insert the transgene into the shuttle plasmid and validate, 3 days for BAC recombineering, DNA preparation and transfection into PK15-IE180 cells and 4–6 days to produce and validate high titer viral neurostocks *in vitro*). However, a few weeks are needed for optimization of injection and validation of labeling *in vivo*.

Although *in vivo* expression of Cre recombinase from IE180-null PRV hSyn-Cre is sufficient to drive recombination in neuronal populations that send even exceedingly sparse projections to the site of injection, expression of fluorescent proteins was either too weak or too transient *in vivo* to drive robust expression in retrogradely labeled neurons from injections in auditory cortex. This contrasts with the observed expression of fluorescent proteins from PRV in cultured neurons more than 30 days after infection. Although the precise reason for this poor expression *in vivo* is unclear, expression levels may be improved in the future by selection of promoters that perform better in the context of the PRV genome as has been done with HSV-1 vectors (Neve et al., 2005).

PRV can be engineered to deliver large transgene cassettes. Up to ~30 kbp can be inserted into the closely related HSV genome without interfering with virus production; a similar capacity is likely for PRV (Reay et al., 2004). This capacity could be exploited to deliver large promoter regions to limit transgene expression to genetically defined cell-types. PRV also holds promise as an alternative to RV for trans-synaptic labeling of neuronal populations. Since the replication and virion production capacity of IE180-null PRV can be rescued by providing IE180 expression *in trans* (e.g., in the PK15-IE180 cell line), this strategy could be applied to enable the transneuronal spread of the virus from anatomically or genetically defined populations of cells to their inputs. Like RV lentivirus and AAV, herpesviruses are amendable to pseudotyping, which may with further development allow for targeting infection to particular cell types (Anderson et al., 2000; Zhou and Roizman, 2005). Moreover, because of its packaging capacity and non-pathogenicity in humans, the use of PRV as a tool in human gene therapy has long been pursued. Replication incompetent, IEG null PRV represents a fundamental advancement towards this goal (Prieto et al., 2002; Boldogköi and Nógrádi, 2003).

Biosafety level (BSL) classification can be an important determinant in the selection of viral tools to be used for a given experiment, as implementation of the appropriate safeguards can be expensive and time consuming. There are no specific official (CDC, NIH, WHO, USDA) references to how replicating PRV should be classified. However, it is reasonable that IE180-null PRV should be classified in the lowest risk group BSL-1. Unmodified PRV does not infect humans, and thus by definition is classified as Risk Group 1 (RG1; National Institutes of Health, 2002; Centers for Disease Control, 2009; World Health Organization WHO, 2014). Concerns about PRV as a biohazard derive from the fact that the wild-type replicating virus is an animal pathogen, although it is not a human pathogen (Pomeranz et al., 2005; Card and Enquist, 2012). Specifically it causes an economically important disease of swine (Kluge et al., 1999). Naturally occurring strains of the virus have the capacity to infect and kill a broad range of mammalian hosts with the important exception of higher primates (Nara, 1982). Furthermore, even the attenuated but replication-competent PRV tracing strains will kill infected animals. Out of general concern for animals, particularly swine, some biosafety committees tend to err on the conservative side by requiring PRV work be treated as BSL-2. The IE180-null PRV described in this manuscript is completely replication incompetent: when stocks are prepared on complementing cells, the virus particles produced are capable of infecting a cell but not of carrying out replication of viral DNA or production of virus particles. The infection cannot spread *in vitro* or *in vivo*. Importantly, IE180-null PRV does not kill animals even when high titer stocks are injected directly into the brain. Therefore, working with IE180-null PRV presents minimal risks to the investigator and animals and as such can reasonably be classified as BSL-1.

In summary, IE180-null PRV recombinants can be constructed that enable the expression of transgenes to facilitate the labeling and functional dissection of anatomically defined cell types. The large genome size of PRV (~143 kb) can accommodate much larger expression cassettes than is possible with other commonly used viral vectors. Unlike its cousin HSV-1, wild-type PRV has a broad host range from some avian species to a number of mammals including Rhesus monkeys and marmosets. However, PRV is not pathogenic in humans, and fewer safety concerns are associated with its application in the laboratory (Kluge et al., 1999; Pomeranz et al., 2005). The family of immediate-early gene deficient PRV vectors described here offers a novel vehicle for gene delivery in the central nervous system.

## Conflict of interest statement

The authors declare that the research was conducted in the absence of any commercial or financial relationships that could be construed as a potential conflict of interest.

## Footnotes

^1^http://goo.gl/dRqh2d

^2^http://goo.gl/J4F4z0
